# Poly[diaqua­bis(2,2′-bipyridine)tris­(μ_4_-2,2′-bipyridine-4,4′-dicarboxyl­ato)dineodymium(III)]

**DOI:** 10.1107/S1600536807067396

**Published:** 2007-12-21

**Authors:** Chia-Jung Tsai, Yen-Hsiang Liu

**Affiliations:** a Department of Chemistry, Fu Jen Catholic University, Taipei 24205, Taiwan

## Abstract

In the crystal structure of the title mixed-ligand coordination polymer, [Nd_2_(C_12_H_6_N_2_O_4_)_3_(C_10_H_8_N_2_)_2_(H_2_O)_2_]_*n*_, the Nd^III^ ion is in an octa­hedral coordination environment formed by one water mol­ecule, one chelating 2,2′-bipyridine ligand, and five monodentate carboxyl­ate groups. The local coordination polyhedron around the Nd^III^ ion is a bicapped trigonal prism. Two Nd^III^ centers are bridged by four carboxyl­ate groups to form an Nd_2_ dimeric unit; these are further connected by 2,2′-bipyridine-4,4′-dicarboxyl­ate linkers, resulting in a layered coordination network.

## Related literature

Only lanthanide-2,2′-bipyridine-4,4′-dicarboxyl­ate-based coordination polymers with three-dimensional porous framework structures have been previously reported (Wu *et al.*, 2006 ▶).
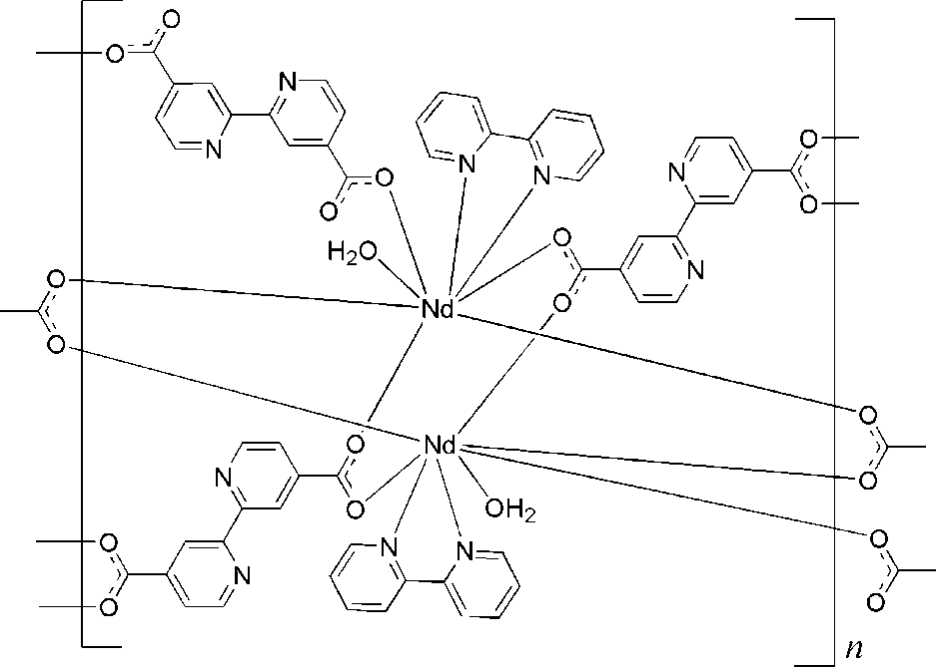


## Experimental

### 

#### Crystal data

[Nd_2_(C_12_H_6_N_2_O_4_)_3_(C_10_H_8_N_2_)_2_(H_2_O)_2_]
*M* *_r_* = 681.72
Triclinic, 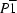

*a* = 8.9914 (2) Å
*b* = 12.5409 (3) Å
*c* = 12.7455 (3) Å
α = 67.809 (2)°
β = 88.645 (2)°
γ = 75.437 (1)°
*V* = 1283.89 (6) Å^3^
*Z* = 2
Mo *K*α radiation
μ = 2.08 mm^−1^
*T* = 200 (2) K
0.5 × 0.4 × 0.2 mm

#### Data collection

Nonius KappaCCD diffractometer
Absorption correction: multi-scan (*SORTAV*; Blessing, 1995 ▶) *T*_min_ = 0.398, *T*_max_ = 0.660
4669 measured reflections
16411 independent reflections
4460 reflections with *I* > 2σ(*I*)
*R*_int_ = 0.042

#### Refinement

*R*[*F* ^2^ > 2σ(*F* ^2^)] = 0.022
*wR*(*F* ^2^) = 0.061
*S* = 1.17
4669 reflections
371 parameters
H-atom parameters constrained
Δρ_max_ = 0.56 e Å^−3^
Δρ_min_ = −0.97 e Å^−3^

### 

Data collection: *COLLECT* (Nonius, 2000 ▶); cell refinement: *SCALEPACK* (Otwinowski & Minor 1997 ▶); data reduction: *DENZO* (Otwinowski & Minor 1997 ▶) and *SCALEPACK*; program(s) used to solve structure: *SHELXS97* (Sheldrick, 1997 ▶); program(s) used to refine structure: *SHELXL97* (Sheldrick, 1997 ▶); molecular graphics: *ORTEP-3 for Windows* (Farrugia, 1997 ▶); software used to prepare material for publication: *WinGX* (Farrugia, 1999 ▶).

## Supplementary Material

Crystal structure: contains datablocks global, I. DOI: 10.1107/S1600536807067396/ww2111sup1.cif

Structure factors: contains datablocks I. DOI: 10.1107/S1600536807067396/ww2111Isup2.hkl

Additional supplementary materials:  crystallographic information; 3D view; checkCIF report

## Figures and Tables

**Table:** 

Nd1—O5	2.386 (2)
Nd1—O3^i^	2.399 (2)
Nd1—O4^ii^	2.4120 (19)
Nd1—O1	2.4309 (19)
Nd1—O2^iii^	2.4443 (19)
Nd1—O7	2.5126 (20)
Nd1—N1	2.618 (2)
Nd1—N2	2.659 (2)
O1—C11	1.252 (3)
O2—C11	1.256 (3)
O3—C22	1.255 (4)
O4—C22	1.252 (3)
O5—C23	1.267 (4)
O6—C23	1.246 (4)

**Table:** 

O5—Nd1—O4^ii^	85.16 (7)
O5—Nd1—O1	85.06 (7)
O3^i^—Nd1—O1	81.99 (7)
O4^ii^—Nd1—O1	71.54 (7)
O3^i^—Nd1—O2^iii^	72.10 (7)
O4^ii^—Nd1—O2^iii^	83.53 (7)
O5—Nd1—O7	73.07 (7)
O4^ii^—Nd1—O7	70.18 (7)
O2^iii^—Nd1—O7	69.04 (7)
O5—Nd1—N1	82.69 (7)
O3^i^—Nd1—N1	84.74 (7)
O2^iii^—Nd1—N1	85.45 (7)
O7—Nd1—N1	73.68 (7)
O5—Nd1—N2	72.54 (7)
O3^i^—Nd1—N2	69.59 (7)
O1—Nd1—N2	78.68 (7)
N1—Nd1—N2	61.78 (7)

Symmetry codes: (i) 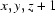; (ii) 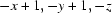; (iii) 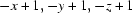.

**Table 2:** Hydrogen-bond geometry (Å, °)

*D*—H⋯*A*	*D*—H	H⋯*A*	*D*⋯*A*	*D*—H⋯*A*
O7—H7*A*⋯O6	0.83	1.92	2.731 (3)	164
O7—H7*A*⋯O5	0.83	2.53	2.918 (3)	110
O7—H7*B*⋯O6^iv^	0.81	2.02	2.811 (3)	165

Symmetry code: (iv) 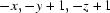.

**Fig. 1.**
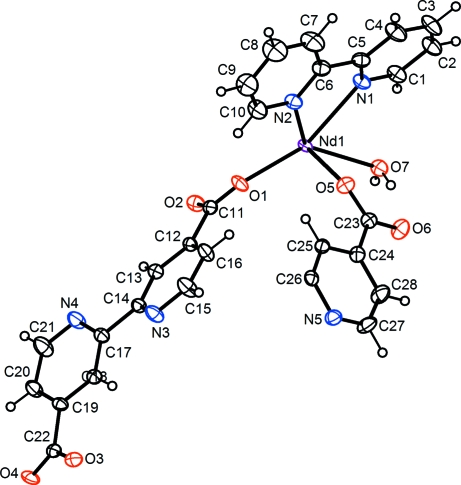
The atom numbering scheme of the title compound presented in the crystallographically asymmetric unit. The atomic displacement ellipsoids are drawn at the 50% probability level.

**Fig. 2.**
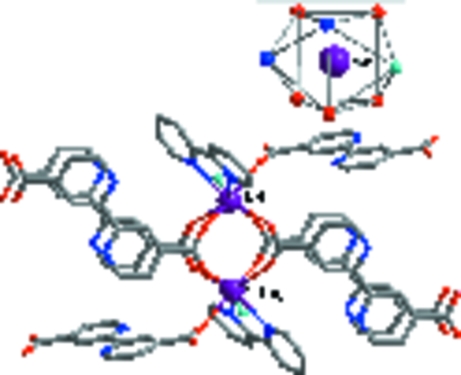
Highlight of coordination environment of the Nd(III) ions. (Key: blue: N; red: O; gray: C; auqa sphere: water molecules; purple sphere: Nd). Hydrogen atoms are omitted for clarity. (Symmetry code: (i) 1–x, 1–y, 1–z) Inset: a view of the bicapped trigonal prism polyhedron around NdIII ion.

**Fig. 3.**
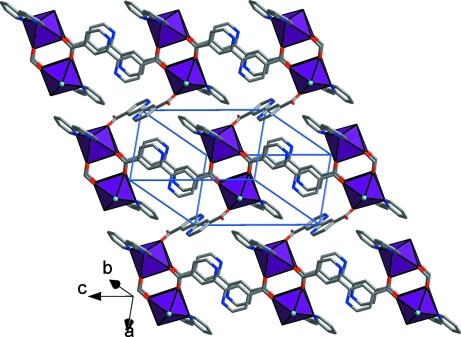
A view of the layer network of the title compound. (Key: auqa sphere: water molecules; purple polyhedra: Nd) Hydrogen atoms are omitted for clarity.

## supplementary crystallographic information

### Comment

As shown in figure 1, a Nd^III^ cation, one 2,2'-bipyridine (bpy) ligand, one coordinated water molecule, as well as one and a half of the 2,2'-bipyridine-4,4'-dicarboxylate (bpdc) ligands were observed in the crystallographic asymmetric unit. The observation of symmetrical C?O bond lengths range from 1.252 (3) Å to 1.267 (4) Å indicated that all of the carboxyl groups of the bpdc ligands are deprotonated to achieve charge neutrality with the Nd^III^ cation. The formula of the title compound is assigned to be [Nd_2_(C_10_H_8_N_2_)_2_(C_12_H_6_N_2_O_4_)_3_(H_2_O)_2_]_n_.

The Nd^III^ ion exists in an eight-coordinated environment formed by one water molecule, one chelating bpy ligand, and five monodentate carboxylate groups of the bpdc ligands. The local coordination geometry around Nd^III^ ion is a bicapped trigonal prism polyhedron. Two Nd^III^ centers are bridged by four carboxylate groups to become a Nd_2_-dimer unit (Figure 2). An inversion center is located at the center of the Nd···Nd axis.

As shown in Figure 3, the Nd_2_-dimer unit is linked through the bpdc ligands to form a layered coordination network. The bpdc ligands present two distinct types of bridging coordination environments, a bis(monodentate) mode as well as a bis(*syn*,*syn*-bridging bidentate) mode. Due to the high oxophilicity (hard acid-hard base interaction) of the lanthanide ions, the bipyridine group of the bpdc ligand is not involved in coordination. Two of the bis(*syn*,*syn*-bridging bidentate) type bpdc bridging ligands are stacked in a parallel fashion. However, no obvious π–π interactions between the inter-ligand pyridine rings are observed (centroid-to-centroid distance of 4.04 Å and dihedral angle of 19.89°). Both of the hydrogen atoms of the coordinated water molecule O7 serve as hydrogen-bonding donors to form two intra-layer and one inter-layer hydrogen-bonding interactions with the oxygen atoms of the carboxylate groups. It is worthwhile noting that the inter-layer O–H···O hydrogen-bonding interactions play an import role in the stabilization of the layer-to-layer stacking in the crystal structure of the title compound. In addition, several intra-layer C–H···O interactions are also observed for the title compound, despite their weakness.

Solely bpdc-based lanthanide coordination polymers with three-dimensional porous framework structures were previously reported (Wu *et al.*, 2006). The diversity in structure dimensionality between the title compound and the compounds reported by Wu *et al.* may be attributed to the presence of chelating bpy ligands in the title compound that capped the connectivity of Nd^III^ ions, and leads to a two-dimensional layered network. Further investigations on mixed-ligand coordination modes with lanthanide ions toward the control of structure topology and network dimensionality are in progress.

### Experimental

All reagents and solvents were used as obtained without further purification. Nd(NO_3_)_3_.6H_2_O (0.30 mmol, 131.7 mg), 2,2'-bipyridine (0.40 mmol, 62.5 mg), 2,2'-bipyridine-4,4'-dicarboxylic acid (0.15 mmol, 36.8 mg) were dissolved in 5.0 ml of distilled water. The mixture was sealed in a Teflon-lined stainless steel vessel and held at 453 K for 96 h. The vessel was gradually cooled to room temperature, and violet crystals suitable for crystallographic analysis were obtained in the yield of 65% based on 2,2'-bipyridine-4,4'-dicarboxylic acid.

### Refinement

The C-bound H atoms were placed in calculated positions (C—H = 0.93 Å) and refined in the riding-model approximation with *U*_iso_(H) = 1.2 *U*_eq_(C). The N-bound H atoms were observed in a difference Fourier map, but were placed in calculated positions (N—H = 0.86 Å) and refined in the riding-model approximation with *U*_iso_(H) = 1.2 *U*_eq_(N). The H atoms of the coordinated water molecules were located in a difference Fourier map, and refined as riding model with *U*_iso_(H) = 1.5 *U*_eq_(O). A longer Nd(1)—O(7) distance of 2.513 (2) Å is observed may be attributed to the loosely bound water molecule.

### Crystal data

**Table:**

[Nd_2_(C_12_H_6_N_2_O_4_)_3_(C_10_H_8_N_2_)_2_(H_2_O)_2_]	*Z* = 2
*M**_r_* = 681.72	*F*_000_ = 676
Triclinic, *P*1	*D*_x_ = 1.763 Mg m^−^^3^
Hall symbol: -P 1	Mo *K*α radiation λ = 0.71073 Å
*a* = 8.9914 (2) Å	Cell parameters from 12726 reflections
*b* = 12.5409 (3) Å	θ = 2.0–25.4º
*c* = 12.7455 (3) Å	µ = 2.08 mm^−^^1^
α = 67.809 (2)º	*T* = 200 (2) K
β = 88.645 (2)º	Prism, white
γ = 75.437 (1)º	0.5 × 0.4 × 0.2 mm
*V* = 1283.89 (6) Å^3^	

### Data collection

**Table:**

Nonius KappaCCD diffractometer	4669 independent reflections
Radiation source: sealed tube	4460 reflections with *I* > 2σ(*I*)
Monochromator: graphite	*R*_int_ = 0.042
*T* = 200(2) K	θ_max_ = 25.3º
φ and ω scans	θ_min_ = 2.4º
Absorption correction: multi-scan(SORTAV; Blessing, 1995)	*h* = −10→10
*T*_min_ = 0.398, *T*_max_ = 0.660	*k* = −14→15
16411 measured reflections	*l* = −15→15

### Refinement

**Table:**

Refinement on *F*^2^	H-atom parameters constrained
Least-squares matrix: full	*w* = 1/[σ^2^(*F*_o_^2^) + (0.0265*P*)^2^ + 1.2551*P*] where *P* = (*F*_o_^2^ + 2*F*_c_^2^)/3
*R*[*F*^2^ > 2σ(*F*^2^)] = 0.022	(Δ/σ)_max_ = 0.002
*wR*(*F*^2^) = 0.061	Δρ_max_ = 0.56 e Å^−^^3^
*S* = 1.17	Δρ_min_ = −0.97 e Å^−^^3^
4669 reflections	Extinction correction: SHELXL97 (Sheldrick, 1997), Fc^*^=kFc[1+0.001xFc^2^λ^3^/sin(2θ)]^-1/4^
371 parameters	Extinction coefficient: 0.0065 (5)

### Special details

**Table:**

Geometry. All e.s.d.'s (except the e.s.d. in the dihedral angle between two l.s. planes) are estimated using the full covariance matrix. The cell e.s.d.'s are taken into account individually in the estimation of e.s.d.'s in distances, angles and torsion angles; correlations between e.s.d.'s in cell parameters are only used when they are defined by crystal symmetry. An approximate (isotropic) treatment of cell e.s.d.'s is used for estimating e.s.d.'s involving l.s. planes.

### Fractional atomic coordinates and isotropic or equivalent isotropic displacement parameters (Å^2^)

**Table:**

	*x*	*y*	*z*	*U*_iso_*/*U*_eq_	
Nd1	0.302446 (16)	0.648646 (11)	0.499494 (11)	0.01470 (8)	
O1	0.4250 (2)	0.69456 (18)	0.32145 (16)	0.0229 (5)	
O2	0.6003 (2)	0.53012 (18)	0.32891 (16)	0.0235 (5)	
O3	0.5424 (2)	0.65947 (18)	−0.43231 (16)	0.0223 (4)	
O4	0.7188 (3)	0.49332 (18)	−0.41903 (17)	0.0241 (5)	
O5	0.0710 (2)	0.77124 (17)	0.38374 (17)	0.0225 (4)	
O6	−0.1170 (3)	0.68020 (19)	0.39864 (18)	0.0284 (5)	
O7	0.0896 (2)	0.55410 (18)	0.58438 (17)	0.0233 (4)	
H7A	0.0142	0.5913	0.5371	0.035*	
H7B	0.1141	0.4849	0.592	0.035*	
N1	0.1807 (3)	0.7494 (2)	0.6388 (2)	0.0215 (5)	
N2	0.3019 (3)	0.8746 (2)	0.4497 (2)	0.0223 (5)	
N3	0.4653 (3)	0.7593 (2)	−0.0901 (2)	0.0258 (6)	
N4	0.7976 (3)	0.5136 (3)	−0.0406 (2)	0.0291 (6)	
N5	−0.1566 (3)	0.9404 (2)	−0.0166 (2)	0.0234 (5)	
C1	0.1138 (4)	0.6873 (3)	0.7282 (2)	0.0251 (7)	
H1	0.1275	0.6047	0.7452	0.03*	
C2	0.0265 (4)	0.7370 (3)	0.7965 (3)	0.0312 (7)	
H2	−0.0176	0.6897	0.8598	0.037*	
C3	0.0047 (4)	0.8573 (3)	0.7704 (3)	0.0369 (8)	
H3	−0.0571	0.8947	0.8147	0.044*	
C4	0.0731 (4)	0.9226 (3)	0.6797 (3)	0.0323 (8)	
H4	0.0592	1.0055	0.6611	0.039*	
C5	0.1628 (4)	0.8665 (3)	0.6156 (2)	0.0221 (6)	
C6	0.2415 (4)	0.9319 (3)	0.5180 (3)	0.0235 (6)	
C7	0.2558 (5)	1.0458 (3)	0.4987 (3)	0.0376 (8)	
H7	0.2134	1.0844	0.5481	0.045*	
C8	0.3316 (5)	1.1022 (3)	0.4078 (3)	0.0426 (9)	
H8	0.3426	1.1796	0.3943	0.051*	
C9	0.3913 (5)	1.0454 (3)	0.3366 (3)	0.0382 (8)	
H9	0.4428	1.0828	0.2726	0.046*	
C10	0.3740 (4)	0.9320 (3)	0.3610 (3)	0.0306 (7)	
H10	0.4157	0.8925	0.3121	0.037*	
C11	0.5080 (3)	0.6300 (2)	0.2764 (2)	0.0178 (6)	
C12	0.4949 (3)	0.6758 (2)	0.1476 (2)	0.0173 (6)	
C13	0.5869 (3)	0.6123 (2)	0.0900 (2)	0.0187 (6)	
H13	0.6611	0.5394	0.1309	0.022*	
C14	0.5690 (3)	0.6570 (2)	−0.0282 (2)	0.0187 (6)	
C15	0.3785 (4)	0.8189 (3)	−0.0329 (3)	0.0306 (8)	
H15	0.3048	0.8915	−0.0754	0.037*	
C16	0.3893 (4)	0.7814 (3)	0.0843 (2)	0.0248 (7)	
H16	0.3252	0.8275	0.1206	0.03*	
C17	0.6656 (3)	0.5932 (2)	−0.0944 (2)	0.0193 (6)	
C18	0.6182 (3)	0.6167 (2)	−0.2056 (2)	0.0183 (6)	
H18	0.5249	0.6749	−0.2412	0.022*	
C19	0.7085 (3)	0.5544 (2)	−0.2641 (2)	0.0177 (6)	
C20	0.8448 (4)	0.4715 (3)	−0.2093 (3)	0.0267 (7)	
H20	0.9094	0.4265	−0.2464	0.032*	
C21	0.8844 (4)	0.4559 (3)	−0.0993 (3)	0.0347 (8)	
H21	0.9795	0.4007	−0.063	0.042*	
C22	0.6534 (3)	0.5710 (2)	−0.3818 (2)	0.0182 (6)	
C23	−0.0411 (3)	0.7506 (2)	0.3433 (2)	0.0190 (6)	
C24	−0.0843 (3)	0.8186 (2)	0.2173 (2)	0.0197 (6)	
C25	−0.0014 (3)	0.8963 (2)	0.1525 (2)	0.0189 (6)	
H25	0.082	0.9084	0.187	0.023*	
C26	−0.0414 (3)	0.9566 (2)	0.0364 (2)	0.0181 (6)	
C27	−0.2347 (4)	0.8644 (3)	0.0472 (3)	0.0307 (7)	
H27	−0.3158	0.8519	0.0107	0.037*	
C28	−0.2040 (4)	0.8027 (3)	0.1641 (3)	0.0294 (7)	
H28	−0.2641	0.7508	0.2062	0.035*	

### Atomic displacement parameters (Å^2^)

**Table:**

	*U*^11^	*U*^22^	*U*^33^	*U*^12^	*U*^13^	*U*^23^
Nd1	0.02095 (11)	0.01231 (10)	0.01183 (10)	−0.00483 (6)	0.00305 (6)	−0.00552 (7)
O1	0.0345 (12)	0.0225 (11)	0.0154 (10)	−0.0109 (9)	0.0101 (9)	−0.0095 (8)
O2	0.0321 (12)	0.0209 (11)	0.0154 (10)	−0.0086 (9)	0.0020 (8)	−0.0036 (8)
O3	0.0259 (11)	0.0226 (11)	0.0192 (10)	−0.0087 (9)	−0.0010 (8)	−0.0073 (8)
O4	0.0366 (12)	0.0228 (11)	0.0216 (11)	−0.0120 (9)	0.0075 (9)	−0.0157 (9)
O5	0.0247 (11)	0.0169 (10)	0.0236 (11)	−0.0065 (8)	−0.0019 (8)	−0.0043 (8)
O6	0.0287 (12)	0.0233 (11)	0.0281 (12)	−0.0128 (9)	0.0017 (9)	−0.0007 (9)
O7	0.0286 (12)	0.0204 (10)	0.0214 (11)	−0.0085 (9)	0.0041 (9)	−0.0075 (8)
N1	0.0286 (14)	0.0169 (12)	0.0195 (13)	−0.0039 (10)	0.0028 (10)	−0.0088 (10)
N2	0.0296 (14)	0.0190 (12)	0.0193 (13)	−0.0070 (11)	0.0024 (10)	−0.0081 (10)
N3	0.0301 (15)	0.0262 (14)	0.0153 (12)	0.0019 (11)	−0.0001 (10)	−0.0074 (11)
N4	0.0280 (15)	0.0361 (16)	0.0196 (13)	0.0024 (12)	−0.0004 (11)	−0.0134 (12)
N5	0.0254 (14)	0.0251 (13)	0.0221 (13)	−0.0107 (11)	0.0017 (10)	−0.0091 (11)
C1	0.0348 (18)	0.0187 (15)	0.0188 (15)	−0.0041 (13)	0.0030 (13)	−0.0057 (12)
C2	0.043 (2)	0.0305 (17)	0.0203 (16)	−0.0108 (15)	0.0096 (14)	−0.0101 (13)
C3	0.047 (2)	0.0352 (19)	0.0346 (19)	−0.0062 (16)	0.0157 (16)	−0.0243 (16)
C4	0.048 (2)	0.0223 (16)	0.0309 (18)	−0.0077 (15)	0.0094 (15)	−0.0160 (14)
C5	0.0270 (16)	0.0174 (14)	0.0233 (15)	−0.0044 (12)	−0.0005 (12)	−0.0101 (12)
C6	0.0285 (16)	0.0194 (15)	0.0231 (15)	−0.0067 (12)	0.0006 (12)	−0.0083 (12)
C7	0.059 (2)	0.0209 (16)	0.038 (2)	−0.0137 (16)	0.0098 (17)	−0.0158 (15)
C8	0.065 (3)	0.0225 (17)	0.045 (2)	−0.0208 (17)	0.0118 (19)	−0.0121 (16)
C9	0.052 (2)	0.0279 (18)	0.036 (2)	−0.0203 (17)	0.0109 (17)	−0.0074 (15)
C10	0.042 (2)	0.0252 (16)	0.0267 (17)	−0.0139 (15)	0.0093 (14)	−0.0090 (13)
C11	0.0227 (15)	0.0181 (14)	0.0152 (14)	−0.0121 (12)	0.0047 (11)	−0.0053 (11)
C12	0.0224 (15)	0.0181 (14)	0.0134 (13)	−0.0103 (11)	0.0049 (11)	−0.0053 (11)
C13	0.0213 (15)	0.0167 (13)	0.0178 (14)	−0.0052 (11)	0.0016 (11)	−0.0060 (11)
C14	0.0206 (14)	0.0207 (14)	0.0160 (14)	−0.0063 (12)	0.0026 (11)	−0.0078 (11)
C15	0.0338 (18)	0.0276 (17)	0.0179 (15)	0.0100 (14)	−0.0024 (13)	−0.0063 (13)
C16	0.0251 (16)	0.0271 (16)	0.0188 (15)	0.0001 (13)	0.0043 (12)	−0.0096 (13)
C17	0.0231 (15)	0.0188 (14)	0.0168 (14)	−0.0064 (12)	0.0034 (11)	−0.0073 (11)
C18	0.0214 (15)	0.0157 (13)	0.0188 (14)	−0.0066 (11)	0.0026 (11)	−0.0066 (11)
C19	0.0252 (15)	0.0154 (13)	0.0158 (14)	−0.0091 (11)	0.0037 (11)	−0.0073 (11)
C20	0.0277 (17)	0.0299 (17)	0.0236 (16)	−0.0023 (13)	0.0046 (13)	−0.0149 (13)
C21	0.0308 (19)	0.039 (2)	0.0249 (17)	0.0097 (15)	−0.0022 (14)	−0.0140 (15)
C22	0.0241 (15)	0.0188 (14)	0.0171 (14)	−0.0145 (12)	0.0057 (11)	−0.0073 (12)
C23	0.0207 (15)	0.0122 (13)	0.0231 (15)	−0.0017 (11)	0.0005 (12)	−0.0070 (11)
C24	0.0207 (15)	0.0157 (13)	0.0227 (15)	−0.0053 (11)	0.0027 (11)	−0.0069 (11)
C25	0.0184 (14)	0.0184 (14)	0.0233 (15)	−0.0055 (11)	0.0028 (11)	−0.0114 (12)
C26	0.0177 (14)	0.0148 (13)	0.0232 (15)	−0.0027 (11)	0.0040 (11)	−0.0097 (12)
C27	0.0308 (18)	0.0354 (18)	0.0309 (18)	−0.0197 (15)	−0.0020 (14)	−0.0110 (14)
C28	0.0313 (18)	0.0287 (17)	0.0282 (17)	−0.0182 (14)	0.0015 (13)	−0.0044 (14)

### Geometric parameters (Å, °)

**Table:**

Nd1—O5	2.386 (2)	C4—H4	0.95
Nd1—O3^i^	2.399 (2)	C5—C6	1.484 (4)
Nd1—O4^ii^	2.4120 (19)	C6—C7	1.394 (4)
Nd1—O1	2.4309 (19)	C7—C8	1.376 (5)
Nd1—O2^iii^	2.4443 (19)	C7—H7	0.95
Nd1—O7	2.5126 (20)	C8—C9	1.376 (5)
Nd1—N1	2.618 (2)	C8—H8	0.95
Nd1—N2	2.659 (2)	C9—C10	1.385 (5)
O1—C11	1.252 (3)	C9—H9	0.95
O2—C11	1.256 (3)	C10—H10	0.95
O2—Nd1^iii^	2.4443 (19)	C11—C12	1.517 (4)
O3—C22	1.255 (4)	C12—C16	1.379 (4)
O3—Nd1^iv^	2.399 (2)	C12—C13	1.392 (4)
O4—C22	1.252 (3)	C13—C14	1.392 (4)
O4—Nd1^ii^	2.4120 (19)	C13—H13	0.95
O5—C23	1.267 (4)	C14—C17	1.494 (4)
O6—C23	1.246 (4)	C15—C16	1.384 (4)
O7—H7A	0.8298	C15—H15	0.95
O7—H7B	0.8078	C16—H16	0.95
N1—C1	1.341 (4)	C17—C18	1.388 (4)
N1—C5	1.351 (4)	C18—C19	1.385 (4)
N2—C10	1.342 (4)	C18—H18	0.95
N2—C6	1.350 (4)	C19—C20	1.386 (4)
N3—C15	1.338 (4)	C19—C22	1.512 (4)
N3—C14	1.344 (4)	C20—C21	1.384 (5)
N4—C21	1.337 (4)	C20—H20	0.95
N4—C17	1.343 (4)	C21—H21	0.95
N5—C27	1.336 (4)	C23—C24	1.512 (4)
N5—C26	1.347 (4)	C24—C28	1.380 (4)
C1—C2	1.376 (4)	C24—C25	1.384 (4)
C1—H1	0.95	C25—C26	1.390 (4)
C2—C3	1.379 (5)	C25—H25	0.95
C2—H2	0.95	C26—C26^v^	1.488 (6)
C3—C4	1.374 (5)	C27—C28	1.391 (5)
C3—H3	0.95	C27—H27	0.95
C4—C5	1.390 (4)	C28—H28	0.95
			
O5—Nd1—O3^i^	141.66 (7)	C6—C7—H7	120.1
O5—Nd1—O4^ii^	85.16 (7)	C9—C8—C7	119.3 (3)
O3^i^—Nd1—O4^ii^	123.72 (7)	C9—C8—H8	120.4
O5—Nd1—O1	85.06 (7)	C7—C8—H8	120.4
O3^i^—Nd1—O1	81.99 (7)	C8—C9—C10	118.0 (3)
O4^ii^—Nd1—O1	71.54 (7)	C8—C9—H9	121
O5—Nd1—O2^iii^	142.08 (7)	C10—C9—H9	121
O3^i^—Nd1—O2^iii^	72.10 (7)	N2—C10—C9	123.9 (3)
O4^ii^—Nd1—O2^iii^	83.53 (7)	N2—C10—H10	118.1
O1—Nd1—O2^iii^	124.64 (7)	C9—C10—H10	118.1
O5—Nd1—O7	73.07 (7)	O1—C11—O2	125.5 (3)
O3^i^—Nd1—O7	136.56 (7)	O1—C11—C12	117.3 (2)
O4^ii^—Nd1—O7	70.18 (7)	O2—C11—C12	117.2 (2)
O1—Nd1—O7	137.04 (7)	C16—C12—C13	118.2 (3)
O2^iii^—Nd1—O7	69.04 (7)	C16—C12—C11	120.5 (3)
O5—Nd1—N1	82.69 (7)	C13—C12—C11	121.3 (2)
O3^i^—Nd1—N1	84.74 (7)	C12—C13—C14	119.2 (3)
O4^ii^—Nd1—N1	143.82 (8)	C12—C13—H13	120.4
O1—Nd1—N1	140.45 (7)	C14—C13—H13	120.4
O2^iii^—Nd1—N1	85.45 (7)	N3—C14—C13	122.8 (3)
O7—Nd1—N1	73.68 (7)	N3—C14—C17	115.7 (2)
O5—Nd1—N2	72.54 (7)	C13—C14—C17	121.5 (3)
O3^i^—Nd1—N2	69.59 (7)	N3—C15—C16	124.1 (3)
O4^ii^—Nd1—N2	144.11 (7)	N3—C15—H15	118
O1—Nd1—N2	78.68 (7)	C16—C15—H15	118
O2^iii^—Nd1—N2	130.91 (7)	C12—C16—C15	118.8 (3)
O7—Nd1—N2	126.03 (7)	C12—C16—H16	120.6
N1—Nd1—N2	61.78 (7)	C15—C16—H16	120.6
C11—O1—Nd1	132.49 (18)	N4—C17—C18	122.9 (3)
C11—O2—Nd1^iii^	152.00 (19)	N4—C17—C14	116.5 (2)
C22—O3—Nd1^iv^	124.74 (17)	C18—C17—C14	120.6 (3)
C22—O4—Nd1^ii^	148.51 (19)	C19—C18—C17	119.3 (3)
C23—O5—Nd1	134.30 (17)	C19—C18—H18	120.3
Nd1—O7—H7A	105	C17—C18—H18	120.3
Nd1—O7—H7B	109.2	C18—C19—C20	118.4 (3)
H7A—O7—H7B	109.9	C18—C19—C22	120.3 (3)
C1—N1—C5	118.2 (3)	C20—C19—C22	121.2 (3)
C1—N1—Nd1	118.68 (19)	C21—C20—C19	118.3 (3)
C5—N1—Nd1	122.42 (19)	C21—C20—H20	120.8
C10—N2—C6	117.7 (3)	C19—C20—H20	120.8
C10—N2—Nd1	120.7 (2)	N4—C21—C20	124.2 (3)
C6—N2—Nd1	121.29 (19)	N4—C21—H21	117.9
C15—N3—C14	116.9 (3)	C20—C21—H21	117.9
C21—N4—C17	116.9 (3)	O4—C22—O3	125.5 (3)
C27—N5—C26	117.3 (3)	O4—C22—C19	117.3 (3)
N1—C1—C2	123.4 (3)	O3—C22—C19	117.1 (2)
N1—C1—H1	118.3	O6—C23—O5	125.5 (3)
C2—C1—H1	118.3	O6—C23—C24	118.5 (3)
C1—C2—C3	118.2 (3)	O5—C23—C24	116.1 (2)
C1—C2—H2	120.9	C28—C24—C25	118.7 (3)
C3—C2—H2	120.9	C28—C24—C23	121.0 (3)
C4—C3—C2	119.4 (3)	C25—C24—C23	120.3 (3)
C4—C3—H3	120.3	C24—C25—C26	119.4 (3)
C2—C3—H3	120.3	C24—C25—H25	120.3
C3—C4—C5	119.6 (3)	C26—C25—H25	120.3
C3—C4—H4	120.2	N5—C26—C25	122.4 (3)
C5—C4—H4	120.2	N5—C26—C26^v^	116.1 (3)
N1—C5—C4	121.2 (3)	C25—C26—C26^v^	121.5 (3)
N1—C5—C6	116.8 (3)	N5—C27—C28	123.8 (3)
C4—C5—C6	122.0 (3)	N5—C27—H27	118.1
N2—C6—C7	121.3 (3)	C28—C27—H27	118.1
N2—C6—C5	116.7 (3)	C24—C28—C27	118.4 (3)
C7—C6—C5	121.9 (3)	C24—C28—H28	120.8
C8—C7—C6	119.8 (3)	C27—C28—H28	120.8
C8—C7—H7	120.1		

Symmetry codes: (i) *x*, *y*, *z*+1; (ii) −*x*+1, −*y*+1, −*z*; (iii) −*x*+1, −*y*+1, −*z*+1; (iv) *x*, *y*, *z*−1; (v) −*x*, −*y*+2, −*z*.

### Hydrogen-bond geometry (Å, °)

**Table:**

*D*—H···*A*	*D*—H	H···*A*	*D*···*A*	*D*—H···*A*
O7—H7A···O6	0.83	1.92	2.731 (3)	164
O7—H7A···O5	0.83	2.53	2.918 (3)	110
O7—H7B···O6^vi^	0.81	2.02	2.811 (3)	165

Symmetry codes: (vi) −*x*, −*y*+1, −*z*+1.
